# Acute Effects of Tissue Flossing Coupled with Functional Movements on Knee Range of Motion, Static Balance, in Single-Leg Hop Distance, and Landing Stabilization Performance in Female College Students

**DOI:** 10.3390/ijerph19031427

**Published:** 2022-01-27

**Authors:** Szu-Ying Wu, Yi-Hsun Tsai, Yu-Ting Wang, Wen-Dien Chang, Chia-Lun Lee, Chun-En Aurea Kuo, Nai-Jen Chang

**Affiliations:** 1 Department of Chinese Medicine, Kaohsiung Chang Gung Memorial Hospital, Kaohsiung 833, Taiwan; rickywu0818@gmail.com (S.-Y.W.); amylilytsai@hotmail.com (Y.-H.T.); 2 School of Chinese Medicine for Post-Baccalaureate, I-Shou University, Kaohsiung 824, Taiwan; 3 Department of Sports Medicine, Kaohsiung Medical University, Kaohsiung 807, Taiwan; jo31724@gmail.com; 4 Department of Sport Performance, National Taiwan University of Sport, Taichung 404, Taiwan; changwendien@gmail.com; 5 Center for Physical and Health Education, National Sun Yat-Sen University, Kaohsiung 804, Taiwan; karenlee1129@gmail.com; 6 Ph.D. Program in Biomedical Engineering, College of Medicine, Kaohsiung Medical University, Kaohsiung 807, Taiwan; 7 Department of Medical Research, Kaohsiung Medical University Hospital, Kaohsiung 807, Taiwan

**Keywords:** exercise, injury prevention, sports performance, myofascial release, flexibility

## Abstract

Flexibility, specifically that in the amplitude of sagittal-plane range of motion (ROM), can improve jump landing patterns and reduce the potential for sports injury. The use of floss bands (FLOSS) reportedly increases joint range of motion (ROM) in the shoulder, ankle, and elbow joints. However, little research on the effectiveness of FLOSS on the knee joint has been conducted. This study investigated the effects of FLOSS on knee ROM, static balance, single-leg-hop distance, and landing stabilization performance in women. This study had a crossover design. Twenty active female college students without musculoskeletal disorders were randomly assigned to receive a FLOSS intervention or elastic bandage (ELA) control on their dominant knees. The participants underwent FLOSS and ELA activities on two occasions with 48 h of rest between both sets of activities. The outcomes were flexibility of the quadriceps and hamstrings, how long one could maintain a single-leg stance (with and without eyes closed), distance on a single-leg triple hop, and score on the Landing Error Scoring System (LESS); these outcomes were evaluated at preintervention and postintervention (immediately following band removal and 20 min later). After the FLOSS intervention, the participants’ hamstring flexibility improved significantly (immediately after: *p* = 0.001; 20 min later: *p* = 0.002), but their quadricep flexibility did not. In addition, FLOSS use did not result in worse single-leg stance timing, single-leg triple-hop distance, or landing stabilization performance relative to ELA use. Compared with the ELA control, the FLOSS intervention yielded significantly better LESS at 20 min postintervention (*p* = 0.032), suggesting that tissue flossing can improve landing stability. In conclusion, the application of FLOSS to the knee improves hamstring flexibility without impeding static balance, and improves single-leg hop distance and landing stabilization performance in women for up to 20 min. Our findings elucidate the effects of tissue flossing on the knee joint and may serve as a reference for physiotherapists or athletic professionals in athletic practice settings.

## 1. Introduction

The knee joint plays a key role in lower-limb biomechanics and is the joint most commonly affected in sports-related injuries of the lower limbs; such injuries are often linked to disabilities of the hip and ankle joints [1]. Knee joint injuries frequently lead to complicated musculoskeletal problems and affect sports performance. Kaeding et al. reported that knee injuries in high school athletes account for 60% of sports-related surgeries [2]. A US study on high-school sports-related injuries that analyzed data from the National High School Sports Related Injury Surveillance System reported an overall rate of 2.98 knee injuries per 10,000 athlete exposures [3]. Among the sports-related injuries analyzed in a 10-year study by Majewski et al., 39.8% were related to the knee joint [4]. Women are two to eight times more likely than men to have certain kinds of knee problems (e.g., anterior cruciate ligament tear). The reasons for the higher rate of knee injuries in women are postulated [5,6]: (1) internal factors such as differences in the anatomical configuration (e.g., higher Q angle), knee ligament, ligament laxity and muscle strength and (2) external factors, such as conditioning, type of training and the development of muscle coordination. Single-limb stabilization ability is typically indicated by the following clinical measures: duration over which one can sustain a single-leg stance, distance reached on a single-leg triple-hop test, and performance in landing stabilization.

Landing stabilization of the lower kinetic chain involves a complex integration of muscle actions that influence the relative positions of the foot, ankle, knee, and hip. When the alignment of one segment of the kinetic chain is altered (resulting in a reduced joint range of motion), the function of other segments is affected [7]. Furthermore, through reductions in peak vertical ground reaction force and dynamic knee valgus load, a greater range of motion (ROM) of the lower limbs may reduce the risk of musculoskeletal injury when the body lands while bearing load [8,9].The Landing Error Scoring System (LESS) is an effective assessment of dual-limb landing stabilization and can help identify the risks of knee injury associated with different landing patterns [10,11,12]. Dynamic knee valgus malalignment can be observed through performance in the jump-landing task [10]. Increased hip adduction, shallow knee flexion, and reduced ankle dorsiflexion are precipitating factors of knee valgus [1,13,14]. Studies have reported that excessive knee valgus during exercise increases the risk of the anterior cruciate ligament (ACL) injury [1,15,16].

Tissue flossing has been used to prevent sports injuries and improve sports performance [17]. Floss bands (FLOSS) are a novel tool used to improve joint ROM [18,19] or reduce pain [20]. They can be applied before or after sports for injury prevention or rehabilitation. Developed by physical therapist Kelly Starrett [21], FLOSS is a type of elastic band made of rubber that can be wrapped around joints or muscle groups during exercise or stretching. Most tissue flossing mechanisms involve blood flow restriction (BFR). Reperfusion, fascial shearing, and occlusion of blood to muscle tissue may be the physiological mechanisms underlying the effect of flossing [22]. For example, tissue flossing results in temporary tissue ischemia and blood flow reperfusion after FLOSS removal, which can enhance metabolic response and alter the microenvironment by increasing growth hormone and catecholamine levels. These hormones may affect sports performance. In addition, blood flow may increase muscle strength and contraction efficiency [23]. Moreover, tissue flossing may increase the pain threshold through compression (e.g., gate control theory), thus improving ROM and muscle flexibility [24,25]. Studies have investigated the application of tissue flossing on the shoulder [21,26,27,28], elbow [29], and ankle joints [18,19,24,25,30]. Many studies involving tissue flossing over the ankle have been conducted and have reported significant increases in ankle dorsiflexion [18,19,24,31]; according to these studies’ findings, these increases were sustainable [19] and were still present at least 7 h after the removal of FLOSS [31]. Improvements in other parameters, such as velocity of the single-leg vertical jump test and jump and sprint performance, have also been observed [18,19]. However, conflicting outcomes have been observed in weight-bearing lunge tests [18,24,30]. Several studies have demonstrated other clinical uses of tissue flossing, such as treating delayed onset muscle soreness after exercise [32], reducing postoperative lower-limb pedal edema [33], and improving pain and movement in chronic Achilles tendinopathy [28]. Studies have yet to thoroughly explore whether a combination of tissue flossing and functional exercises for the knee joint can improve sports performance. Marco et al. investigated the effect of tissue flossing on perceived knee pain and vertical jump performance in five young male recreational athletes with knee pain [20]. Significant differences in vertical jump performance and perceived pain were identified between the tissue flossing protocol and the nonflossing protocol. In addition, Maust et al. reported that tissue flossing on hamstrings resulted in greater hamstring flexibility than did a control condition [34]. A recent systematic review and meta-analysis assessed changes in ankle dorsiflexion resulting from the use of FLOSS, but did not further investigate other outcomes such as balance, muscle force output, or functional test performance [25].

The effect of tissue flossing on flexibility and static and dynamic balance in the lower limbs requires further research. The aforementioned outcomes are related to sports performance [35]. Postural stability can be measured through static or dynamic tests, and poor postural stability is a critical risk factor for damage during landing. Balance assessments are performed to evaluate athletes’ risk of injury as well as their potential sports performance [36,37]. Static balance is defined as the maintenance of a stable base of support. Static postural stability can be assessed with an individual maintaining a single-leg stance with the eyes open or closed [38]. However, stability in static balance may not necessarily translate to postural control during dynamic movements because of the task demands of such movements. Therefore, dynamic postural stability can be assessed through tests in which participants change the location of their base of support while maintaining their postural stability, as in a single-leg hop test [39]. Furthermore, jump landing is one of the most frequently performed actions in sports [35]. The landing error scoring system (LESS) is an inexpensive, valid, and reliable clinical tool for assessing jump-landing stabilization to identify athletes with biomechanical patterns presenting a high injury risk [15]. The related measurements are easily collected through real-time screening on the field. Therefore, this study investigated the effect of tissue flossing and functional exercises on knee ROM and sports performance. We hypothesized that among women, the application of FLOSS to the knee joint would increase muscle flexibility without impeding static balance, distance achieved in the single-leg hop test, or landing stabilization performance. The primary outcome was knee ROM because it has been the main variable evaluated in previous FLOSS studies. To further elucidate the effect of the treatment on sports performance, the secondary outcomes measured were static balance, distance in single-leg hop test, and landing stabilization performance.

## 2. Materials and Methods

### 2.1. Participants

The study was approved by Institutional Review Board of Kaohsiung Medical University Hospital (KMUHIRB-F(II)-20190113). Twenty healthy female university students (mean age: 21.8 ± 2.31 years; mean height: 162.2 ± 6.41 cm; mean weight: 55.6 ± 7.84 kg) who were active in recreational sports were enrolled. The participants’ basic information and medical history were collected to screen for eligibility and potential risk factors. Prospective participants were included only if they were women who were at least 20 years old and recreationally active (exercising 2–3 times weekly) [40]. Women were chosen as participants because they are 2 to 8 times more likely than men to have specific types of knee problems [5,9]. The exclusion criterion was the presence of any severe lower-limb (i.e., hip, knee, or ankle) musculoskeletal illness (dislocation, fracture, muscular rupture, tendon rupture, or ligament rupture) or acute injury (sprain, strain, open wound) within 6 months. The study procedures and risks were thoroughly explained to all the participants, and informed consent was obtained from all participants.

### 2.2. Study Procedures

The study adopted a crossover design. Each participant was subject to both the intervention and control. Specifically, each participant was randomly assigned to condition A or condition B (randomization was performed using the Random Team Generator program, https://www.randomlists.com, accessed on 28 November 2021) to determine whether the elastic bandage (ELA) control or FLOSS intervention was to be administered first. The two trials were performed separately 48 h apart [40]. During this interval, participants were prohibited from participating in intense exercise, which was defined by a rating of perceived exertion greater or equal to 14 [41] and included activities such as weight training and moderate- to high-intensity running. All the procedures (elastic bandage (ELA) wrapping, tissue flossing using FLOSS, and personalized functional exercise guidance) were performed by one sports medicine professional to avoid any further injury or muscular compensation. Before each trial, the participants were asked to perform a stationary bike warm-up program for 5 min to prevent exercise injury [42]. All the experiments were performed in a laboratory of the Department of Sports Medicine at Kaohsiung Medical University. The assessments were performed immediately after and 20 min after each intervention. The order of tests for all participants was as follows: knee ROM, single-leg stance, single-leg triple hop, and LESS. The FLOSS or ELA was removed after the participants completed all of the exercise-related tasks. Postintervention measurements were conducted in the same order as the preintervention measurements were. 

### 2.3. Outcome Measures

Before initiating data collection, the researchers received comprehensive training on how all outcome measurements were to be collected. 

#### 2.3.1. Primary Outcome

The primary outcome was muscle flexibility. An Ely test was used to assess the flexibility of each patient’s rectus femoris [43]. This test has a high intraclass correlation coefficient (ICC = 0.91) [44]. During assessment, each patient lay prone in a relaxed state. The sports medicine professional set the axis of the goniometer to the femoral epicondyle of the tested leg and aligned the stationary arm with the greater trochanter of the femur and aligned the moving arm with the lateral malleolus of the fibula. We subsequently asked the patient to flex her knee as much as possible and measured the knee angle until the patient’s hip lifted off the table. Generally, if the hip flexes prematurely, the rectus femoris is tight. Adequate flexibility enables the knees to flex to 120° [45]. In this study, the ICC of this assessment was 0.915, which indicated the excellent reliability of the measures.

A popliteal angle test was used to assess the flexibility of each patient’s hamstrings [46]. The patient lay supine in a relaxed state, and the sports medicine professional held the tested hip and knee in a 90° flexion position. The axis of the goniometer was placed at the femoral epicondyle of the tested leg. The sports medicine professional then aligned the stationary arm vertical to the floor and the moving arm with the lateral malleolus of the fibula. The participant was asked to actively extend their knee as much as possible. The popliteal angle was measured under these conditions. In this study, the ICC of this assessment was 0.8525, which indicated the excellent reliability of the measures.

#### 2.3.2. Secondary Outcomes

The secondary outcomes were time spent sustaining a single-leg stance [47], distance on the single-leg triple-hop test [48], and landing stabilization performance [49]. A 30 s single-leg stance test was used to assess each patient’s static balance when their eyes were open and when they were closed. The test began once the nontested foot was lifted off the floor with the participant’s arms akimbo, and it ended when the nontested foot dropped to the floor, the body shook, or the arm stretched out due to loss of balance. The test was terminated once the maximum duration of 30 s had elapsed. A longer duration indicates greater static balance. In this study, the ICCs of this assessment were 0.843 and 0.088 for the tests with the eyes closed and eyes open, respectively.

A single-leg triple-hop test was used to assess each participant’s single-leg dynamic balance. The sports medicine professional fixed a piece of tape on the ground that was perpendicular to a starting line as the standard measure point. The participant stood on the tested leg with their hallux (big toe) on the starting line. They performed three consecutive maximal hops forward on the same leg. The physical therapist measured the hopping distance from the starting line to the participant’s heel after the participant landed the third hop. Right (R) and left legs were both tested twice, and the maximum distance (in cm) achieved during the two trials was recorded. In this study, the ICC of this assessment was 0.979.

A landing stabilization test was used to assess the risk of knee injury and abnormal knee valgus [15,16]. The jump-landing task incorporated vertical and horizontal movements. Each participant jumped down from the top of a 30-cm-high box to a marked point on the floor that was 50% of the participant’s height away from the box. The participants were asked to immediately rebound as high as possible upon reaching the marked point on the floor. Two consumer-grade video cameras captured the slow-motion frontal and sagittal plane view of each participant during the testing procedure, and the participant’s landing stabilization performance was scored using the LESS [49]. The total possible score on the LESS is 19 (Table 1). The first 15 items of the LESS are used to evaluate the movement of the individual’s knee, hip, and trunk, and proper and improper motion are indicated by scores of 0 and 1, respectively. The two global items reflect overall joint movement and the general perception of landing quality. Good, normal, and improper motion are given scores of 0, 1, and 2, respectively. In general, scores < 5 indicate *good* landing quality, which is associated with a low risk of ACL injury [15]. In this study, the LESS was reliable at an ICC of 0.889.

### 2.4. Intervention Protocols

#### 2.4.1. Floss Band Intervention

**Figure 1:**
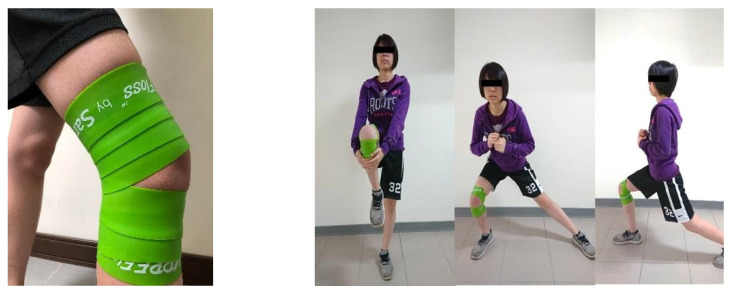
Floss Band Intervention.

The sports medicine professional wrapped FLOSS around the dominant knee of each participant according to the instructions of Sanctband, the manufacturer, and guided the participant to perform the following functional movement tasks: walking knee hugs, side squats, and forward lunges [46]. Each participant was required to perform 10 repetitions of each task over 3 min at a tempo of 30 beats per minute [46] according to a metronome (https://stonekick.com/metronome.html, accessed on 28 November 2021). The floss band used in this study (Figure 1) was COMPRE Floss, which was developed by Sanctband in collaboration with physiotherapist Sven Kruse. A 5 cm-wide green floss band was employed in this study. We followed the wrapping technique suggested by the Sanctband user manual. In brief, while the participant stood and performed slight knee flexion, the band was wrapped upward from the tibial tuberosity on the participant’s dominant side as the starting point to 5 cm above the femoral epicondyle. Care was taken to ensure the patella remained uncovered. Pressure was produced through wrapping of the joint with 50% tension and 50% overlap. The wrapping method is displayed on the Sanctband official website (https://www.sanctband.com/comprefloss-flossband/, accessed on 28 November 2021).

#### 2.4.2. Elastic Bandage Control

As a control, a 4-inch ELA was wrapped around the dominant knee of each participant with the same wrapping technique as for the floss band, and the physical therapist guided the participant to perform the same three functional movement tasks that they performed when wearing the FLOSS wrapping [50]. The ELA, when applied in accordance with manufacturer instructions, is designed to stretch in accordance with the movement of the body for comfort. The band tension was produced by overlapping half of the previous part of the band, distally to proximally. The participants again performed 10 repetitions of each movement tasks in 3 min [31] at a tempo of 30 beats per minute, as measured using the same metronome.

### 2.5. Statistical Analyses

The requisite minimum sample size of 20 was calculated a priori based on the anticipated differences in knee extension ROM as the primary outcome. The anticipated mean difference in the FLOSS group was 4° with a standard deviation of 6° between preintervention and immediately postintervention. The calculation was also based on an alpha level of 0.05 and a desired statistical power of 80% using G*Power software [51].

Statistical analyses were performed using SPSS software (V. 21.0, SPSS, Chicago, IL, USA). The level of statistical significance was set to *p* < 0.05 for all analyses. All data are presented in terms of the mean ± standard deviation. Descriptive statistics were used to analyze participant characteristics, such as age, height, and weight. A Shapiro–Wilk test (*p* > 0.05) was used to evaluate the normality of the data, and the homogeneity of variance was verified using Levene’s test. If the sphericity assumption was violated in Mauchly’s sphericity test, the Greenhouse–Geisser adjustment was used for corrections in the degrees of freedom. A 2 (condition: FLOSS vs. ELA) × 2 (time: preintervention vs. immediately postintervention vs. 20 min postintervention) repeated measures analysis of variance (ANOVA) was performed to evaluate the effects of all measured variables. One-way ANOVA with a Bonferroni-adjusted post hoc test was conducted if a significant main effect was identified. 

## 3. Results

All the participants completed all of the trials in the study without adverse events. The results of all outcomes are presented in Table 2, Table 3 and Table 4. No significant differences in preintervention measurements were identified between the two conditions. 

### 3.1. Primary Outcomes

In our analysis of quadricep flexibility, no significant condition × time interaction was observed (F = 1.697, *p* = 0.207), and the main effects of condition (F = 2.110, *p* = 0.163) and time (F = 2.712, *p* = 0.079) were nonsignificant (Table 2).

In our analysis of hamstring flexibility, a significant condition × time interaction was observed (F = 6.295, *p* = 0.004), which indicated a difference in group and time interventions. The main effect of time was statistically significant (F = 5.540, *p* = 0.008), but the main effect of the condition was not. The post hoc measures reflected improvements in hamstring flexibility immediately after (*p* = 0.001) and 20 min after (*p* = 0.002) the FLOSS intervention, relative to the preintervention measurements. 

### 3.2. Secondary Outcomes

In our analysis of static balance, no significant condition × time interaction was observed, and the main effects of time and condition were nonsignificant. 

As indicated in Table 3, in our analysis of dynamic balance, the effects of the FLOSS intervention on single-leg triple hop for both feet did not exhibit a significant condition × time interaction (HopR: *F* = 0.665, *p* = 0.520; HopL: *F* = 1.138, *p* = 0.330) or significant main effects for time (HopR: *F* = 3.430, *p* = 0.060; HopL: *F* = 1.892, *p* = 0.175) or condition (HopR: *F* = 0.007, *p* = 0.935; HopL: *F* = 0.459, *p* = 0.560). 

In our analysis of landing stabilization, no significant condition × time interaction was observed (*F* = 0.650, *p* = 0.528; Table 4). However, the main effects of time (*F* = 4.632, *p* = 0.016) and condition (*F* = 4.453, *p* = 0.048) were significant. The improvement at 20 min postintervention (*F* = 5.325, *p* = 0.032) was significantly higher in the FLOSS group than in the ELA group, which suggests that the participants exhibited better landing stability at 20 min postintervention after tissue flossing than after application of the ELA.

## 4. Discussion

This is the first study to explore the acute effects of FLOSS on knee ROM, static balance, single-leg hop distance, and landing stabilization performance. The results of our study indicated that after receiving a FLOSS applied to the knee joint, the participants exhibited significantly higher hamstring flexibility for up to 20 min. The participants exhibited no significant changes in static or dynamic balance. We also observed between-group differences in LESS 20 min after the removal of the FLOSS or ELA. In addition, none of the participants reported pain, numbness, cold or hot sensations, skin irritation, or other adverse effects during or after the mechanical compression and transient BFR in this study. Thus, tissue flossing with FLOSS may be a safe method through which young women can attenuate hamstring tightness and improve landing stabilization, and can be applied in further studies of the lower limbs in sports performance.

The possible therapeutic effects of floss bands may be attributed to a combination of myofascial rehydration, partial vascular occlusion, and local BFR [18,19,52,53]. The compression of FLOSS and the movements performed while flossing induce fascial shearing, which can deform adhesion points, facilitate myofascial sliding, and facilitate the restoration of normal fascial alignment [24]. Improved joint ROM and kinetic chain mobility can be obtained once the gliding potential of the fascia is restored and the excessive pressure on the trigger point is removed. The compression during flossing causes local BFR and affects the treatment site in several respects. First, the compression of a local site may reduce the influx of inflammatory mediators and thereby reduce the inflammatory response and sensitivity of nociceptors [32]. Second, the compression of a floss band and the additional functional movements trigger mechanoreceptors, according to the gate control theory of pain, which results in pain relief [54]. Finally, reactive reperfusion occurs after flossing and induces changes in local microenvironments. The relative enhanced blood flow nourishes the muscle and facilitates the metabolism of intramuscular byproducts [24]. Reperfusion to the temporary ischemic area has been reported to induce the release of exercise performance-related factors, such as growth hormones and catecholamines, and alter muscle contraction strength and torque [19,23,55,56,57].

The results of this study revealed that wrapping FLOSS around the knee and performing functional movements can significantly improve hamstring flexibility. The average popliteal angle increased by 3.7% from preintervention to immediately after flossing and increased by 2.8% 20 min postintervention. This finding is similar to that of another study, in which flossing over the knee in recreationally active men without musculoskeletal disorders significantly improved the flexibility of the quadriceps and hamstrings, as measured using Ely’s test and a popliteus test [46]. Moreover, the results of the present study revealed that tissue flossing could improve joint ROM up to 20 min after FLOSS removal. However, the participants’ quadriceps’ flexibility did not improve significantly after flossing. This was probably because most of the participants exhibited tightening in the hamstrings [58], with an average popliteal angle of 127.60° after the ELA activity and of 127.43° after the FLOSS intervention. By contrast, the participants’ quadricep flexibility levels at baseline were generally normal [45], which resulted in no significant change being observed after tissue flossing. In a study involving tennis players, FLOSS use yielded no significant improvements in elbow ROM among participants with normal elbow ROM but yielded improvements in mean elbow ROM among participants with initially restricted elbow ROM [29]. Similarly, another study reported that active stretching with flossing over an uninjured glenohumeral joint is unlikely to improve soft tissue flexibility [21]. Driller et al. and Mills et al. applied FLOSS over participants’ ankles for 2 min while the participants performed active ROM tasks, and the participants’ jumping and sprinting performance was evaluated using the same measurement in each study. Drills et al. reported significant improvements in the evaluated outcomes; however, Mills et al. did not, and ascribed these inconsistent results to differences in participant characteristics. The groups exhibiting superior baseline conditions or individuals who received more training may have exhibited less potential for performance improvement relative to recreational groups [19,30].

In the static balance and single-leg hop tests, no significant differences were identified among the participants’ values at pretest versus immediately or 20 min after intervention in either group; this indicates that tissue flossing cannot significantly affect the stability and balance of the lower limbs. The improvement of joint ROM might not have been reflected as improvements in complex movements. Studies have also agreed with the viewpoint that results of simple physical tests poorly reflect an individual’s actual performance. Driller et al. observed improvements in ankle ROM and jump and sprint performance after tissue flossing. However, Schache et al. reported that passive ROM tests do not reflect active ROM used in coordinated sports, such as running, due to the complexity of the biological underpinnings of human bodily movement [59]. Yuktasir et al. determined that although 6-week stretching exercise programs increased ROM, they had little effect on the participants’ performance of whole-body movements (e.g., jumping) [60]. Further research is required to assess the benefits of tissue flossing on body movements and motor control. Moreover, whether long-term training with tissue flossing is effective for improving motor control requires further investigation through observational studies.

The LESS can be used to identify dangerous movement patterns associated with ACL injury. LESS scores that are >6 and either 5 or 6 indicate high and moderate risk, respectively [49]. However, conflicting evidence regarding the use of LESS as a predictor of ACL injury has been reported. The participants’ average LESS scores did not decrease significantly immediately after flossing, which indicates that mechanical compression and transient BFR during flossing does not affect the risk of knee injury. Furthermore, at 20 min postintervention, the participants who had received the FLOSS intervention exhibited significantly lower LESS scores than did those who received the ELA control, which indicates that tissue flossing may result in a lower risk of lower-limb injury during landing. We postulated that this result may affect changes in the neuromuscular properties (e.g., electromyographic signal), and even muscle strength after wearing FLOSS. In Konrad’s study, the flossing treatment showed a positive effect on the maximum voluntary contraction of the knee extensors [61]. In addition, in Chang’s study, after FLOSS intervention, quadriceps muscle force output (immediately and 20 min later) was significantly improved [46]. However, whether the effect of flossing on LESS score exhibits a delayed onset and how long this effect lasts for warrant further study.

Few studies have investigated tissue flossing over the knee, and no study has analyzed differences between men and women. In García-Luna’s study, five young male recreational athletes with previously reported knee pain due to patellofemoral pain syndrome performed countermovement jumps with and without FLOSS wrapping over the knee on two separate days [20]. The study concluded that flossing can reduce perceived knee pain and improved vertical jump performance in young male recreational athletes. However, it was limited by its small sample size, and the short interval between the pre- and post-flossing measurements meant that only the immediate effect of tissue flossing was evaluated. Each individual’s patellofemoral syndrome-related pain intensity was not assessed, and the outcome assessment conducted using a visual analogue scale was simplistic and subjective. According to a study by Medeiros et al., joint ROM generally decreases with age, but women exhibit greater joint ROM than men do, regardless of age [62,63]. However, women are more likely to exhibit knee valgus when landing. A smaller knee flexion angle and greater ground reaction force at the moment of landing potentially increase the risk of knee injury [64,65]. Thus, we recruited women as participants in this study to account for the effects of these sex-related characteristics.

This study has some limitations. First, research on tissue flossing has yet to reach maturity as a subfield, and most studies have focused on the shoulder and ankle joints [18,19,27,30]. The effects of flossing on different joints and their underlying mechanisms have not yet been determined. Second, the underlying pressure on the target tissue after flossing was not measured, and the percentage of blood restriction was not assessed using a laser Doppler flowmeter. Third, the participants in this study were all healthy young women. The effects of tissue flossing in populations with knee-related musculoskeletal injures and the differences in these effects between sex or age groups require further investigation. Thus, the generalizability of the results to other populations (e.g., people with musculoskeletal disorders, recreational athletes, and elite athletes) is low, but due to ethical considerations, we did not include a no-treatment control condition in the study. Furthermore, the compression materials (i.e., FLOSS and ELA) employed in this study are different. FLOSS is made of latex rubber, whereas ELA is made of cotton and elastic yarn. The different bands have different structures and levels of elasticity, which may affect the BFR of the targeted tissues and the corresponding physiological effects. Fifth, we did not perform motion analysis to assess whether the use of FLOSS can help to control knee valgus. However, tissue flossing of the thigh could stimulate the vastus lateralis, which could reduce the muscle imbalance between the vastus medialis and vastus lateralis [61], thereby decreasing the knee valgus angle and risk of knee injury. Sixth, we observed that the recreationally active women in this study exhibited tightness of the hamstring and relative flexibility of the quadriceps. The improvements achieved through FLOSS wrapping may be more favorable for individuals with tightness in the hamstrings. In addition, although the change in hamstring flexibility was statistically significant, the clinical significance of the change should be examined.

In practical applications, tissue flossing serves as a new option for athletes who wish to increase their joint ROM quickly and briefly. The program employed in study included three functional movements (walking knee lifts, side squats, and lunges) with 10 repetitions over 3 min at 30 beats per minute. Through the application of the appropriate technique, a few minutes of compression with functional movements can enhance joint ROM without adverse effects. For individuals with poor flexibility, tissue flossing may also be used to restore joint ROM and improve individuals’ performance of daily activities.

## 5. Conclusions

The findings of this study indicate that the use of FLOSS combined with functional movements in female college students can improve hamstring flexibility for up to 20 min and improve landing stabilization for at least 20 min after removal, without impeding static or dynamic balance.

## Figures and Tables

**Table 1:** The Operational Definitions of Error in Landing Error Scoring System Items, Reprinted from ref. [49].

Landing Error Scoring System Item	Operational Definition of Error	Scoring
Knee flexion: initial contact	The knee is flexed less than 30° at initial contact.	0 = Absent
		1 = Present
Hip flexion: initial contact	The thigh is in line with the trunk at initial contact.	0 = Absent
		1 = Present
Trunk flexion: initial contact	The trunk is vertical or extended on the hips at initial contact.	0 = Absent
		1 = Present
Ankle plantar flexion: initial contact	The foot lands heel to toe or with a flat foot at initial contact.	0 = Absent
		1 = Present
Medial knee position: initial contact	The center of the patella is medial to the midfoot at initial contact.	0 = Absent
		1 = Present
Lateral trunk flexion: initial contact	The midline of the trunk is flexed to the left or the right side of the body at initial contact.	0 = Absent
		1 = Present
Stance width: wide	The feet are positioned greater than shoulder width apart (acromion processes) at initial contact.	0 = Absent
		1 = Present
Stance width: narrow	The feet are positioned less than shoulder width apart (acromion processes) at initial contact.	0 = Absent
		1 = Present
Foot position: external rotation	The foot is externally rotated more than 30° between initial contact and maximum knee flexion.	0 = Absent
		1 = Present
Foot position: internal rotation	The foot is internally rotated more than 30° between initial contact and maximum knee flexion.	0 = Absent
		1 = Present
Symmetric initial foot contact: initial contact	One foot lands before the other foot or one foot lands heel to toe and the other foot lands toe to heel.	0 = Absent
		1 = Present
Knee-flexion displacement	The knee flexes less than 45° between initial contact and maximum knee flexion.	0 = Absent
		1 = Present
Hip-flexion displacement	The thigh does not flex more on the trunk between initial contact and maximum knee flexion.	0 = Absent
		1 = Present
Trunk-flexion displacement	The trunk does not flex more between initial contact and maximum knee flexion.	0 = Absent
		1 = Present
Medial knee displacement	At the point of maximum medial knee position, the center of the patella is medial to the midfoot.	0 = Absent
		1 = Present
Joint displacement	Soft: the participant demonstrates a large amount of trunk, hip, and knee displacement. Average: the participant has some, but not a large amount of, trunk, hip, and knee displacement. Stiff: the participant goes through very little, if any, trunk, hip, and knee displacement.	0 = Soft
		1 = Average
		2 = Stiff
Overall impression	Excellent: the participant displays a soft landing with no frontal plane or transverse-plane motion. Average: all other landings. Poor: the participant displays large frontal plane or transverse-plane motion, or the participant displays a stiff landing with some frontal plane or transverse-plane motion.	0 = Excellent
		1 = Average
		2 = Poor

**Table 2:** Flexibility at preintervention, immediately following band removal and 20 min later.

Outcomes (Degree)	ELA			FLOSS			Post hoc
	Pre	Imm	Post20	Pre	Imm	Post20	
Qua	132.68 (6.81)	133.15 (6.50)	133.15 (8.40)	132.85 (9.66)	135.95 (8.81)	135.80 (6.20)	
Ham	127.60 (9.91)	128.60 (11.77)	127.47 (9.25)	127.43 (6.87)	132.20 * (7.79)	131.00 * (7.66)	FLOSS: Pre < Imm, Pre < Post20

*: significant difference compared with preintervention, *p* < 0.05. ELA = elastic bandage, FLOSS = floss band, Ham = hamstring, Qua = quadriceps, data presented as mean (standard deviation). In the post hoc column, Pre = preintervention, Imm = immediately after intervention, Post20 = 20 min after intervention.

**Table 3:** Static balance and distance of single-leg triple hop preintervention, immediately following band removal and 20 min later.

Outcomes	ELA			FLO		
	Pre	Imm	Post20	Pre	Imm	Post20
OpenR ^a^ (s)	30.00 (0.00)	29.52 (2.16)	30.00 (0.00)	29.28 (2.60)	29.79 (0.94)	30.00 (0.00)
CloseR ^a^ (s)	14.39 (10.47)	16.11 (9.50)	13.72 (10.23)	11.74 (10.34)	13.51 (11.85)	18.88 (11.30)
OpenL (s)	28.62 (5.32)	28.99 (3.49)	29.90 (0.32)	28.71 (4.39)	29.86 (0.63)	30.00 (0.00)
CloseL(s)	13.04 (10.90)	14.91 (10.85)	16.82 (11.38)	13.33 (11.47)	16.17 (12.34)	15.41 (12.35)
HOPR (cm)	366.44 (53.39)	377.20 (61.64)	375.64 (58.43)	371.01 (48.43)	377.03 (55.59)	372.58 (54.00)
HOPL (cm)	365.58 (52.41)	374.68 (62.34)	372.94 (53.11)	366.93 (45.45)	371.63 (51.29)	363.15 (47.73)

*p* < 0.05. ELA = elastic bandage, FLOSS = floss band, data presented as mean (SD). Pre = preintervention, Imm = immediately after intervention, Post20 = 20 min after intervention. ^a^ = static balance when their eyes were open and when they were closed. HOP = single-leg triple-hop test. R = right, L = left.

**Table 4:** LESS scores at preintervention, immediately following band removal and 20 min later.

Outcomes (Score)	ELA			FLO			Post hoc
	Pre	Imm	Post20	Pre	Imm	Post20	
LESS	3.65 (1.98)	3.25 (2.00)	3.15 (1.98)	3.38 (2.49)	2.75 (1.80)	2.25 ^#^ (1.68)	Post20: ELA > FLO (*p* = 0.032)

#: significant difference compared between conditions, *p* < 0.05. ELA = elastic bandage, FLOSS = floss band, data presented as mean (standard deviation). In the post hoc column, Pre = preintervention, Imm = immediately after intervention, Post20 = 20 min after intervention.

## Data Availability

Data are contained within the article.
